# Exploring the control of whole-body angular momentum in young and elderly based on the virtual pivot point concept

**DOI:** 10.1098/rsos.240273

**Published:** 2024-09-25

**Authors:** Vahid Firouzi, Omid Mohseni, Andre Seyfarth, Oskar von Stryk, Maziar A. Sharbafi

**Affiliations:** ^1^ Lauflabor Locomotion Laboratory, Institute of Sport Science, TU Darmstadt, Darmstadt, Germany; ^2^ Simulation, Systems Optimization and Robotics Group, Department of Computer Science, TU Darmstadt, Darmstadt, Germany; ^3^ Department of Electrical Engineering and Information Technology, Measurement and Sensor Technology Group, TU Darmstadt, Darmstadt, Germany

**Keywords:** balance control, angular momentum, virtual pivot point, elderly, gait

## Abstract

While walking, ground reaction forces point from the centre of pressure to the neighbourhood of a focal point, namely the virtual pivot point (VPP), that adjusts angular momentum around the centre of mass (CoM). This study explores how age and speed affect the VPP quality and position during walking. Analysing an experimental dataset reveals high quality of the VPP in the sagittal plane for both young and elderly groups, regardless of speed. However, in the frontal plane, the VPP quality decreases with increasing speed, with elderly participants exhibiting significantly lower quality. Although not a direct measure of balance, VPP quality reflects changes in whole-body angular momentum owing to ageing and speed. Additionally, a template model is used to reproduce the VPP quality and position trends observed in the experiment. Simulation results highlight the sensitivity of VPP quality to leg force feedback and show that changing VPP height has minimal effect on gait speed. Furthermore, energy redistribution occurs through increased hip extension and leg damping, associated with a greater horizontal VPP distance from the CoM, observed in elderly walking. This study shows promise for analysing gait based on VPP, potentially aiding clinical interventions and supporting locomotion in the elderly.

## 1. Introduction

Bipedal walking, a fundamental activity in daily human life, involves a sophisticated interplay between the central nervous and the neuromusculoskeletal system. The central nervous system must coordinate and continuously adjust multiple degrees of freedom in the musculoskeletal body to maintain stability and an upright posture while walking. Despite the remarkable adaptability and coordination of the human body, various factors can jeopardize balance and lead to falls [1]. Research indicates that the ageing process can have a significant impact on the neuromuscular balance control system [2,3]. It is reported that approximately one-fourth of United States residents aged 65 years and over (older adults) report falling each year [4]. Impaired postural control stands out as a crucial factor contributing to these falls [5]. Another important factor is the gait speed, which has been shown to affect step width variability [6] and, thus, increase lateral gait unsteadiness. Comparison of the gait between young and elderly adults is one approach to understanding the underlying mechanisms of gait and motor control. This comparative analysis sheds light on age-related changes in gait characteristics and gait deficits that are more prevalent in older adults. Understanding these differences becomes paramount in developing targeted interventions and rehabilitation strategies for individuals with age-related gait impairments, ultimately fostering healthier and more functional ageing.

The impacts of ageing and gait speed on balance have been studied by analysing spatiotemporal parameters such as the extrapolated centre of mass (CoM) [7] and step width [6], which are based on kinematic measurement. However, kinematic parameters alone do not fully account for the interaction forces between the body and the environment, leading to a limitation as a dynamic balance measure. In order to gain a comprehensive understanding of balance mechanics, it is necessary to examine how the ground reaction force (GRF) acts at the centre of pressure (CoP) to alter the body’s angular momentum with respect to its CoM [8]. While the magnitude of angular momentum can vary depending on factors such as walking speed, body mass and individual characteristics, it is the coordinated control of angular momentum that ensures the body’s postural balance during walking gait.

Experimental studies have shown that young, healthy humans effectively direct the GRF to coincide at a point above the CoM—named virtual pivot point (VPP)—to regulate the pattern of angular momentum during walking [9]. The concept of VPP is also observed in human running [10], as well as the gait of some animals such as dogs [9,11], macaques [12] and quails [13]. A recent study investigated the VPP concept in human walking with altered CoP, such as using a rigid sole, walking on stilts or walking barefoot or backwards [14]. Some studies also compared the VPP concept between pathological (e.g. Down syndrome and Parkinson’s disease) and healthy gait [15,16]. However, most of these studies considered VPP in the sagittal plane. The concept of VPP is additionally observed in the frontal plane during walking and running gaits [10,17]. Notably, maintaining frontal plane balance during walking has proved to be more challenging compared to sagittal plane balance [18,19], necessitating active control [20]. Given that irregular regulation of the angular momentum during human walking is linked to an increased risk of falling [21,22], it becomes imperative to comprehend the mechanisms by which humans control their angular momentum. This understanding is crucial for informing the design of assistive devices or interventions aimed at preventing falls [23]. Hence, our study focuses on a comparative analysis of the mechanisms governing angular momentum regulation based on the VPP in both the sagittal and frontal planes within the young and elderly populations.

In this study, our primary objective is to explore the impact of ageing and speed on the quality (how the GRFs coincide at a point) and position of the VPP. We hypothesize that the VPP mechanism for regulating angular momentum differs between young and elderly individuals, particularly in the frontal plane. To accomplish this, we analyse an experimental dataset encompassing 24 healthy young and 18 elderly individuals [24], enabling us to analyse the VPP in both the sagittal and frontal planes. Furthermore, we employ a simulation template model to replicate the observed experimental findings. The simulation model complements the experimental findings by providing insights into potential causal relationships and fostering a deeper comprehension of the underlying mechanisms that drive the experimental outcomes. By integrating empirical data and simulation, our study aims to enhance our understanding of the mechanisms of balance with potential applications in the design of assistive devices [25].

## 2. Method

In this section, we begin by presenting an overview of the experimental dataset pertaining to walking that was employed for our analysis. We then proceed with an explanation of the VPP calculation, along with its assessment in terms of goodness for steady-state walking gaits in both the sagittal and frontal planes. To validate the findings, we introduce a three-dimensional template walker model and elucidate the control strategy used to produce a stable walking pattern.

### 2.1. Experimental dataset

To investigate the VPP during walking of young and elderly subjects, we used motion capture data obtained from the publicly available dataset provided by Fukuchi *et al*. [24]. The dataset comprises kinematic and dynamic treadmill walking recordings of 42 individuals, including 24 young adults (mean age: 27.6±4.4 years, height: 171.1±10.5 cm, mass: 68.4±12.2 kg) and 18 older adults (mean age: 62.7±8.0 years, height: 161.8±9.5 cm, mass: 66.9±10.1 kg). All participants had no lower-extremity injuries within six months prior to data collection, and they were free from any neurological or orthopaedic conditions that could affect their gait patterns. Each subject walked for 90 s under eight different gait-speed conditions, ranging from 40% to 145% of their self-selected dimensionless speed, with the order of conditions randomized. It is worth noting that some elderly subjects did not complete all eight gait-speed conditions (five subjects). Further details about the dataset can be found in the study in [24]. We used raw marker trajectories and GRFs from the dataset. The raw data underwent low-pass filtering with a cut-off frequency of 15 Hz using a fourth-order Butterworth filter.

### 2.2. Computing measures

To determine the VPP, we plotted the GRF vectors from the CoP in a CoM-centred coordinate frame aligned with the vertical in the sagittal plane [26,27] and a CoM-centred coordinate frame aligned with the pelvis orientation in the frontal plane [17]. In our analysis, the dataset we used solely contained lower limb markers, thus necessitating the estimation of CoM position and pelvis orientation using anterior and posterior iliac markers [28,29]. To determine the VPP location, we first identify a line where the angular momentum around each point during the single support phase is constant and equal to the angular momentum around the CoM. The VPP is a point on this line that minimizes the sum of the squared angular momentum [9]. To assess the agreement between experimentally measured GRFs and the ideal VPP model, we employed the coefficient of determination ($R^{2}$) as a quantitative measure [30]:


(2.1) $$R^{2}=\left(1-\frac{\sum_{k=1}^{N_{t}}\sum_{k=1}^{N_{s}}(\theta_{exp}^{ij}-\theta_{VPP}^{ij})}{\sum_{k=1}^{N_{t}}\sum_{k=1}^{N_{s}}(\theta_{exp}^{ij}-{\bar{\theta }}_{exp})}\right)\times \%100,$$

in which $(\theta_{exp}^{ij}$ and $\theta_{VPP}^{ij})$ represent the force vector angles measured at the jth instant of the gait cycle for the ith trial in the experimental data and VPP model-predicted data, respectively. The VPP model predicts the force vector from the CoP to the VPP. $N_{t}$ denotes the number of trials, and $N_{s}$ denotes the number of samples in each trial. In an ideal scenario where all GRFs intersect at a single focused point, achieving $R^{2}=100\%$ indicates a perfect match between the experimental GRF and the theoretical force vector angles. Conversely, a very small $R^{2}$ value suggests that the estimation using VPP is nearly identical to using the mean value ${\bar{\theta }}_{exp}$. The $R^{2}$ value can vary from −Inf to 100%. In our study, we define a 80% threshold as the minimum validity for the VPP concept in data analysis [30,31]. When GRF vectors align closely, $R^{2}$ yields a large negative value. To address this, we treated negative $R^{2}$ values as zero to counteract the impact of outliers, particularly large negative values, on mean value computations. Additionally, this approach aids in presenting results more clearly in figures.

### 2.3. Statistical analysis

We employed a linear mixed-effects model (LMM) to analyse the relationship between the VPP variable (quality and position) and several predictors: age group, speed and dominant leg. Fixed effects include the main effects of age group, speed and dominant leg. To assess the combined impacts of these variables on the response, we also considered analysing their two-way interactions using another LMM. In each of these formulations, we incorporated a random intercept for each subject to account for the individual variability and improve model accuracy. The false discovery rate correction is used to control the expected proportion of false discoveries among all the rejected null hypotheses when conducting multiple comparisons or tests. A significance threshold of 0.05 for *p*-values was chosen to determine significant differences.

### 2.4. Simulation model

**Figure 1.**
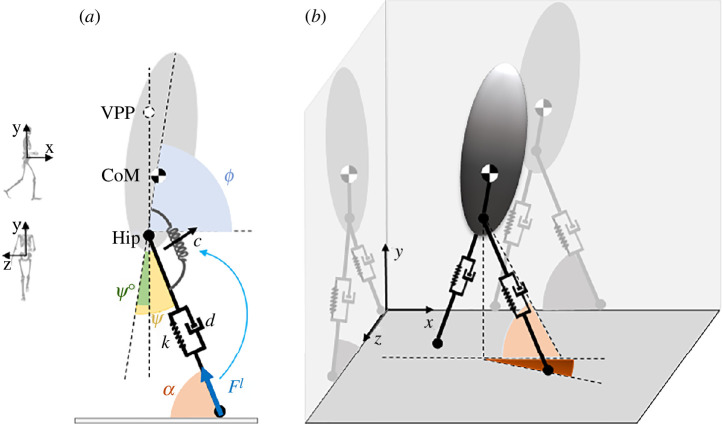
(*a*) Bipedal spring-loaded inverted pendulum model with a rigid trunk and a massless spring for the leg. This figure illustrates the variables and parameters essential for deriving the equations of motion. (*b*) Our developed three-dimensional model. The coordinate system used in this study is shown in the graphics on the left.

In this section, we describe a template model that we developed to gain a deeper understanding of experimental observations. We developed a three-dimenesional template model by extending a three-dimensional spring-loaded inverted pendulum model with the additional trunk. This model consists of two massless springs representing the virtual legs beside a rigid trunk for the upper body with mass m and moment of inertia J (see figure 1). In our model, we defined a virtual hip joint in the middle of the left and right hip joints. The virtual leg is a segment that connects the virtual hip to the foot. Trunk orientation, defined by a line connecting the hip to the CoM, is characterized by the corresponding angles in the sagittal ($\phi_{s}$), frontal ($\phi_{f}$) and horizontal ($\phi_{h}$) planes. By controlling hip torque in the sagittal and frontal planes and choosing an appropriate leg adjustment strategy, stable walking can be achieved in three-dimensional space. The equations of motion and the comparison between human experiment and our model are presented in appendix A.

### 2.5. Control

The control of the model is divided into two distinct phases: stance and swing. During the stance phase, the control system generates hip torques ($\tau_{si}$ and $\tau_{fi}$) using force-modulated compliant hip (FMCH) [32] controller between each leg i and the trunk. It is important to note that controlling the hip torque in the sagittal and frontal planes is sufficient for achieving stable walking. The hip torques of leg i in each plane p are determined by the following equations:


(2.2) $$\tau_{pi}=c_{p}\;F_{pi}^{l}\;(\psi_{pi}-\psi_{pi}^{0})\;p=s,f\;i=1,2,$$

where c denotes the normalized stiffness of the hip springs, $\psi^{0}$ defines the rest angle of the hip springs and ψ corresponds to the angle between the trunk and the leg, as depicted in figure 1*a*. Additionally, $F^{l}$ represents the leg force projection on each plane. This controller enables us to investigate the impact of sensory information and VPP position on gait. The controller parameters are chosen to generate a VPP 18cm above the CoM in the sagittal plane and 5cm above the CoM in the frontal plane, based on experimental data.

During the swing phase, the determination of the leg direction solely focuses on the touch-down moment since the legs in our model are assumed to be massless. To achieve this, we adopt the velocity-based leg adjustment approach, where the leg direction is determined by calculating a weighted average of the CoM velocity vector and the gravity vector [33].

## 3. Results

In this section, we start by delving into the experimental dataset and investigating the VPP in the context of walking for both the young and elderly groups at different speeds. We examine the quality of the VPP, represented by the coefficient of determination ($R^{2}$), and VPP position with respect to the CoM. Subsequently, we use a three-dimensional walking template model to simulate the observed results and determine the significant factors required to replicate human walking outcomes.

### 3.1. Experimental data analysis

#### 3.1.1. Virtual pivot point formation

**Figure 2.**
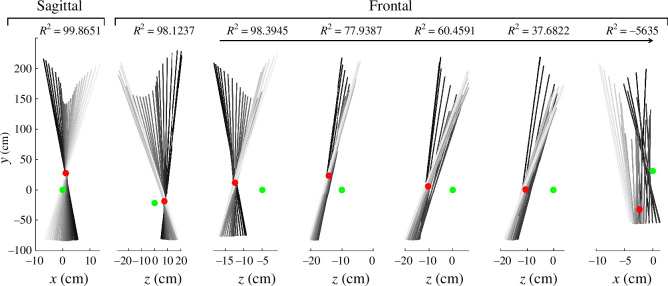
Examples of the intersection of GRF vectors and the calculated VPP for both sagittal (the leftmost figure) and frontal planes during walking experiments. GRFs are represented by solid lines, transitioning from black to grey, indicating the start and end of the single support phase. The second and third figures from the left depict the formation of VPP for the left and right legs, respectively, in the frontal plane. Starting from the third figure and moving towards the end, the subsequent figures portray various instances of VPP with distinct $R^{2}$ values, indicating a spectrum of correlation strength ranging from high to low. Green and red circles show the position of CoM and VPP, respectively. These samples are derived from various subjects to provide an overview of the varied appearances of VPP based on the $R^{2}$ value.

To illustrate the formation of the VPP resulting from the intersections of GRF vectors, figure 2 showcases the VPP in both the sagittal and frontal planes for the right and left legs during preferred walking speed. It also depicts different instances of VPP with varying $R^{2}$ values, demonstrating a range from high to low correlations. High $R^{2}$ values indicate the convergence point of the GRF vectors, forming a singular point, whereas low or negative $R^{2}$ values indicate less coherent formation of the VPP.

#### 3.1.2. Virtual pivot point quality

**Figure 3.**
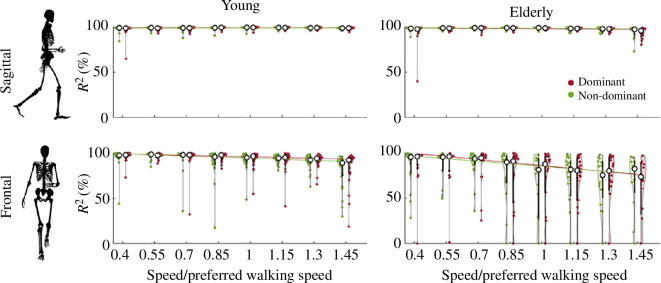
Coefficient of determination ($R^{2}$) for the VPP in sagittal and frontal planes for both groups of young (first column) and elderly (second column) with respect to the normalized walking speed – 0.4 to 1.45 (normalized to the preferred walking speed of each subject). Red and green circles represent the results for the dominant and non-dominant legs, respectively, with each circle indicating the mean value for one subject. The white circles denote the median values for each condition. Solid lines represent the linear fit on the median values.

To quantitatively evaluate the precision of the intersection of GRF vectors at a single point, we examine the coefficient of determination ($R^{2}$). Figure 3 presents the $R^{2}$ coefficient for both the young and elderly groups in the sagittal and frontal planes, considering both the dominant and non-dominant legs. The numerical value for this figure can also be found in the table 1. A comparison between the young and elderly groups reveals that the $R^{2}$ coefficient is higher in the young subjects than in the elderly group, regardless of the plane (see tables 1 and 2). However, this difference is much higher in the frontal plane compared to the sagittal plane (estimated coefficient −1.21 in the sagittal plane versus −19.14 in the frontal plane; see table 2). Gait speed has a significant negative impact on the quality of VPP, and its impact is much higher in the frontal plane compared to the sagittal plane (estimated coefficient −0.3 in the sagittal plane versus −17.2 in the frontal plane; see table 2). The dominant leg also shows a significant negative effect in the both sagittal and frontal planes, suggesting a lower $R^{2}$ value for the dominant leg compared to the non-dominant leg. However, the impact of the dominant leg is minimal compared to the age group and gait speed. The interaction terms indicate more reduction in $R^{2}$ for the elderly group by increasing speed in both the sagittal and frontal planes.

**Table 1.** VPP quality (*R^2^*) and VPP position (*x*, *y*, *z*) in both sagittal and frontal planes for both age groups. (The subscripts D and N are used to denote the dominant and non-dominant legs, respectively. To calculate the values reported in this table, we first calculated the mean for each subject. Then, we computed the median and the interquartile range. The medians are shown in bold font, with the 25th and 75th percentiles presented in parentheses.)

			normalized speed							
plane	group	VPP par.	0.4	0.55	0.7	0.85	1	1.15	1.3	1.45
		$R_{D}^{2}(\%)$	98.3 (96.4, 98.7)	98.3 (97.2, 99.2)	98.5 (96.6, 99.4)	99 (98.4, 99.4)	99 (98.5, 99.4)	98.8 (98.2, 99.4)	98.8 (98.2, 99.3)	98.4 (97.6, 99.1)
		$R_{N}^{2}(\%)$	98.7 (98, 99)	98.8 (97.7, 99.1)	98.5 (98, 99.1)	98.8 (98.2, 99.3)	99.1 (98.5, 99.4)	98.9 (98.3, 99.3)	98.6 (98, 99.3)	98.4 (97.5, 99)
		$y_{D}(cm)$	33.6 (27.6, 54.5)	33.3 (27.7, 45.8)	33.5 (25.7, 48.9)	31.5 (26.1, 40.1)	25.8 (21.3, 33)	25.1 (18.7, 30.2)	21.4 (16.3, 27.2)	19.9 (11.9, 24.4)
	young	$y_{N}(cm)$	31.2 (24, 43.3)	34.9 (25, 43)	38.9 (25.9, 47.5)	31 (24, 40.1)	26.1 (21.1, 34.6)	25 (20.3, 32.8)	24.3 (18.5, 30.7)	22.5 (15.6, 24.7)
		$x_{D}(cm)$	2.7 (1.6, 4.2)	2.4 (1.3, 3.8)	2.1 (1.3, 3.8)	2 (1.3, 3.5)	1.6 (1, 3.4)	1.8 (0.4, 3.5)	1.4 (0.4, 3.1)	1.5 (0.4, 3.6)
		$x_{N}(cm)$	3.8 (2.1, 4.9)	3.6 (2.2, 4.9)	3.4 (1.7, 4.6)	3.3 (1.8, 4.3)	2.9 (1.4, 4.2)	2.8 (1.2, 3.7)	2.5 (1, 3.9)	2.5 (1, 3.8)
sagittal		$R_{D}^{2}(\%)$	97.3 (95, 98.7)	98.1 (95.7, 98.7)	98.6 (97.7, 99)	98.5 (97.8, 98.8)	98.4 (97.2, 98.9)	97.8 (97.1, 98.6)	97.5 (95.1, 98.4)	95.6 (88.5, 97.9)
		$R_{N}^{2}(\%)$	97.3 (95, 98.7)	98.2 (95.7, 98.7)	98.6 (97.7, 99)	98.5 (97.8, 98.8)	98.4 (97.2, 98.9)	97.8 (97.1, 98.6)	97.5 (95.1, 98.5)	95.6 (88.5, 97.9)
		$y_{D}(cm)$	43.3 (31.5, 47.2)	41.3 (30.9, 49.6)	38.9 (34, 48.9)	37.8 (27, 42.9)	32.5 (23.5, 40)	26 (23.2, 34.8)	24.8 (20, 29.8)	20.7 (17.6, 23)
	elderly	$y_{N}(cm)$	44 (34.1, 51.6)	39.2 (32.2, 52.3)	37 (31.6, 43.6)	31.6 (29.1, 38.5)	31.4 (26.2, 37.5)	27.8 (22, 34)	29.5 (20, 33.8)	26.8 (18, 34.3)
		$x_{D}(cm)$	4.2 (2.9, 5.9)	3.7 (3.2, 6.5)	3.6 (2.3, 6)	3.6 (3.3, 5.8)	3.6 (2.7, 5.6)	3.5 (2.8, 5.3)	3.2 (2.8, 4.7)	3.4 (2.7, 5.2)
		$x_{N}(cm)$	4.2 (1.7, 5)	4.2 (2.6, 5.5)	4 (2.1, 5.2)	3.7 (1.6, 5.3)	3.8 (1, 5)	3.4 (0.7, 5)	3.8 (1, 5.8)	4.1 (−3, 5.8)
		$R_{D}^{2}(\%)$	97.6 (95.5, 98.9)	97 (95.5, 98.2)	97.6 (95.6, 98.4)	97.5 (93.5, 98.7)	96 (90.6, 97.6)	94.7 (90.6, 97.8)	93.7 (89, 95.7)	91.8 (77.8, 94.9)
		$R_{N}^{2}(\%)$	97.1 (96, 99)	98.1 (95.3, 98.6)	97.6 (96.4, 98.9)	95.5 (88, 97.9)	94.6 (89.3, 97)	94 (90.7, 96.5)	92.1 (88.4, 95.5)	88.7 (74.6, 92.7)
		$y_{D}(cm)$	−0.8 (−5.4, 6.2)	1.2 (−4.8, 5)	0 (−8, 7.5)	2.5 (−5.5, 11.1)	2.8 (−5.5, 12.6)	2.9 (−4.2, 15.1)	2.2 (−3.6, 13.2)	−0.2 (−6.5, 14.4)
	young	$y_{N}(cm)$	−4.9 (−17, 7.3)	8.6 (6, 12.9)	0.5 (−2, 4.5)	−2 (−6, 4)	0.5 (−3.8, 3.2)	−3.4 (−6.7, 2.1)	−6.6 (−15,–0.8)	5.6 (−0.1,6.6)
		$z_{D}(cm)$	1.1 (−0.1, 1.9)	−0.3 (−0.5, 0.2)	0.2 (−0.3, 0.5)	0 (−0.4, 0.4)	0 (−0.8, 0.5)	1.2 (0.7, 1.6)	−0.3 (−0.6, 0.6)	−1.1 (−1.8, –0.5)
		$z_{N}(cm)$	−4.9 (−16, 7.3)	8.6 (6, 12.9)	0.5 (−2.1, 4.5)	−1.9 (−6, 4)	0.5 (−3.8, 3.2)	−3.4 (−6.7, 2.2)	−6.6 (−15, 0.8)	5.6 (−0.1, 6.6)
frontal		$R_{D}^{2}(\%)$	95.8 (82.7, 96.9)	95.9 (80, 96.4)	94.2 (91.3, 97.6)	90.6 (45.5, 95.3)	87.4 (56.5, 95.8)	80.4 (47.8, 93.9)	80 (58.8, 94.9)	73.5 (33, 87.9)
		$R_{N}^{2}(\%)$	95.4 (78.3, 97.4)	95.3 (80.2, 96.5)	93.4 (87.1, 96.9)	89.6 (67.5, 96.1)	81.3 (29.11, 93.2)	81.5 (53.2, 90.7)	75.3 (51, 90)	82.3 (56.3, 90.3)
		$y_{D}(cm)$	4 (−4.1, 11.3)	9.9 (−3.8, 17.2)	7.8 (−0.2, 15.5)	11 (6.2, 16.3)	12.8 (4.1, 15.5)	8.4 (−6.5, 17.6)	4.2 (0, 12.5)	5.8 (−6, 17.3)
	elderly	$y_{N}(cm)$	3.3 (−3.6, 16.6)	3 (−10, 10.5)	3.4 (−1.8, 10.5)	−0.7 (−4.7, 10.5)	0 (−10.8, 5.8)	−1.7 (−15.9, 3)	−0.2 (−17.5, 7.3)	−0.5 (−4.7, 10.7)
		$z_{D}(cm)$	−0.2 (−2, 1.1)	−0.5 (−1.8, 1.4)	0.1 (−1.2, 1.6)	0.5 (−0.8, 1.2)	0.1 (−1.1, 1.4)	−0.1 (−1, 1.8)	−0.6 (−1.1, 1.3)	−0.9 (−1.9, 1)
		$z_{N}(cm)$	−0.4 (−1.3, 0.9)	−0.7 (−1.5, –0.2)	−0.5 (−1.2, 0.6)	−0.45 (−1.6, –0.1)	−0.4 (−0.9, 1.1)	−0.8 (−1.6, 0.7)	−0.5 (−1.8, 0.7)	−1 (−1.9, –0.1)

**Table 2.** Results of the linear mixed-effects model (LMM) analysis. (Significant relationships are indicated with an asterisk (*), and estimated coefficients are provided in parentheses. The horizontal VPP position in the frontal plane is analysed separately for the right and left legs, as the hip joint is separated by the pelvis in this plane. Subscripts R and L denote the right and left legs, respectively. For categorical variables (age group and dominant leg), the base groups for comparison are elderly (subscript E) and dominant leg (subscript D).

			main effects			interactions	
plane	VPP par.	age_E_	speed	leg_D_	age_E_:speed	age_E_:leg_D_	speed:leg_D_
$R^{2}$	* (−1.21)	* (−0.3)	* (−0.3)	* (−1.93)	* (−0.62)	* (0.2)	
*x*	* (1.5)	* (−1)	* (−0.08)	* (−0.7)	* (1.4)	* (−0.6)	
sagittal	*y*	—	* (−18)	* (−0.8)	* (−14.6)	* (3.8)	* (−4.6)
$R^{2}$	* (−19.14)	* (−17.2)	* (−0.7)	* (−24.4)	—	* (−1.11)	
$z_{R}$	—	—	—	—	—	—	
$z_{L}$	—	* (−0.1)	—	—	—	* (0.3)	
frontal	*y*	—	—	* (3)	* (−7)	* (1.4)	* (3.2)

#### 3.1.3. Virtual pivot point position

**Figure 4.**
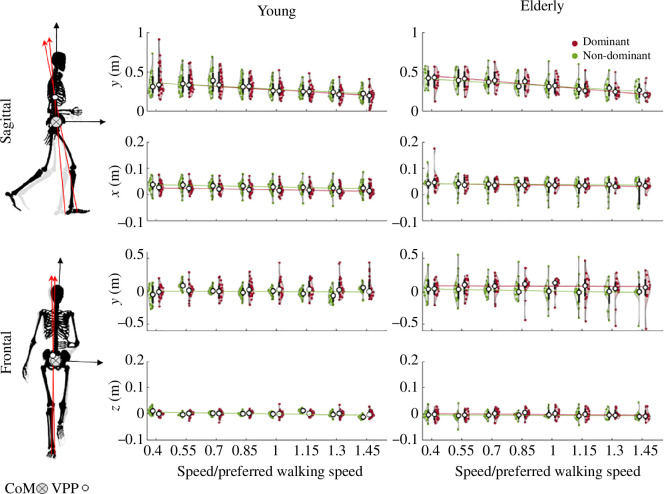
VPP position with respect to the CoM position in the sagittal (x, y) and frontal (z, y) planes for both groups of young (first column) and elderly (second column) with respect to the normalized walking speed – 0.4 to 1.45. Red and green circles represent the VPP position for the dominant and non-dominant legs, respectively, with each circle indicating the mean value for one subject. White circles denote the median VPP positions for steps with $R^{2}>80\%$. Approximately 0.5% and 20% of steps are eliminated based on this threshold in the sagittal and frontal planes, respectively.

Shown in figure 4 are the VPP positions with respect to the CoM position in the sagittal (x, y) and frontal (z, y) planes for both groups of young and elderly. Numerical values for this figure can be found in table 1. In this figure, the VPP positions of those steps with $R^{2}>80\%$ are depicted. The 80% threshold is selected to ensure the presence of valid VPPs.

In the sagittal plane, both the young and elderly groups exhibit VPPs positioned above the CoM and there is no significant difference between age groups. Additionally, it is observed that both groups display a decreasing trend in VPP height as speed increases (see table 2). In the frontal plane, the vertical position of the VPP is significantly related to the dominant leg and is above the CoM for both young and elderly groups at most speeds (see tables 1 and 2). However, the vertical position of the VPP for the non-dominant leg is below the CoM at some speeds, especially for the young group. Turning attention to the mediolateral VPP position (z), there is no significant difference between young and elderly groups (see table 2). In both age groups, the mediolateral VPP is located close to the CoM (see table 1).

### 3.2. Simulation results

Based on the observed experimental results and statistical analyses, there are significant differences in the $R^{2}$ value in both planes and the VPP position in the horizontal direction between the young and elderly groups. Furthermore, there is a significant correlation between forward walking speed and VPP height. Consequently, our objective is to employ our three-dimensional template model to reproduce these findings. This replication effort is undertaken to gain insights into the underlying factors influencing the VPP. By employing the FMCH controller, our model successfully generates stable walking gaits at various speeds. A comparison between the generated gait with our model and human data is depicted in figure 8 in appendix A.

#### 3.2.1. Sensitivity analyses of sensory feedback

**Figure 5.**
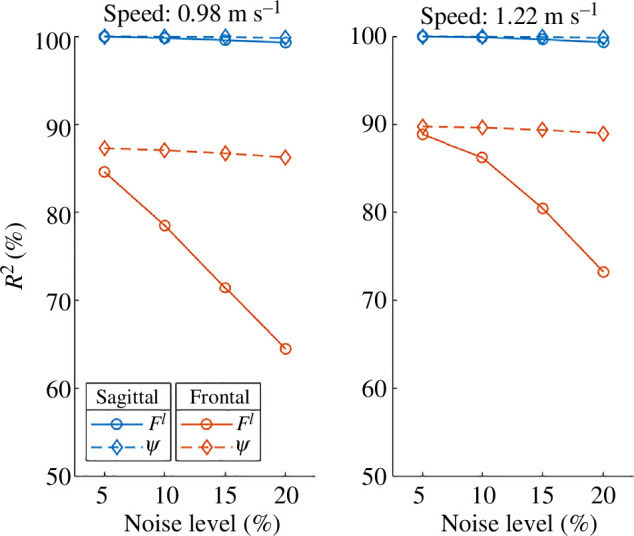
Sensitivity analysis on VPP quality ($R^{2}$) using simulated results. The impact of uniform noise, spanning from 5% to 20% of the magnitude of sensory inputs (leg force $F^{l}$ and angle between the leg and trunk ψ in equation 2.2), on $R^{2}$ is depicted in the sagittal and frontal planes.

To conduct sensitivity analysis, we introduced varying degrees of noise (up to 20%) to the sensory feedback signals ($F^{l}$ and ψ) in our model. These are the sensory feedback we have in our FMCH controller. The results depicted in figure 5 reveal that the quality of VPP is more sensitive to sensory noise in the frontal plane compared to the sagittal plane. Also, leg force is a more sensitive sensory feedback than the angle between the trunk and leg in both the sagittal and frontal planes, displaying a monotonic decline in the focus point quality with increasing noise. Furthermore, VPP quality increases with speed.

#### 3.2.2. Influence of virtual pivot point height on forward walking speed

**Figure 6.**
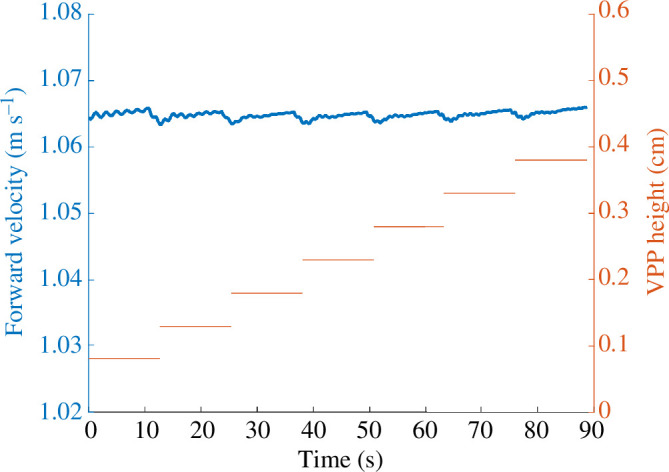
Speed effect of VPP height change on walking speed. The left axis shows the forward velocity of the centre of mass, and the right axis shows the VPP height in the sagittal plane.

Investigations into the VPP position, depicted in figure 4, unveiled a noteworthy pattern: as walking speed increases, there is a significant reduction in the VPP height within the sagittal plane (*y*-direction) (refer to table 2). To replicate this empirically observed correlation, we conducted simulations by manipulating the VPP height and subsequently examined its impact on walking speed. This approach was chosen owing to the VPP position serving as our direct controllable parameter. The outcome of this simulation is illustrated in figure 6, and the results demonstrate that variations in the VPP height (ranging from 8 to 38cm) do not result in substantial alterations to forward walking speed.

#### 3.2.3. Reproducing virtual pivot point position in elderly

**Figure 7.**
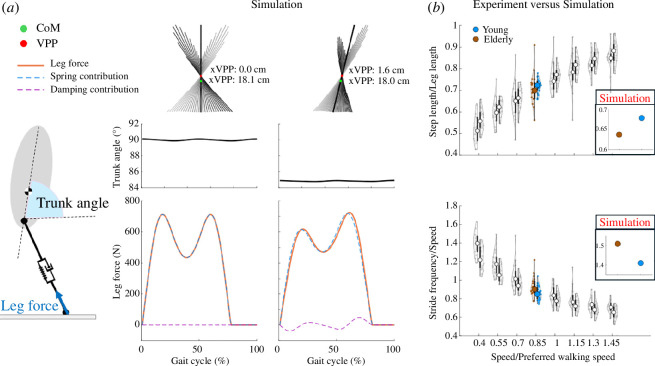
(*a*) Influence of anterior sagittal-plane VPP shift on trunk angle and leg force. Top row: variations in horizontal VPP position lead to distinct patterns of GRF vectors intersecting at the VPP. Middle row: changes in trunk angle in response to the VPP shift. Bottom row: analysis of spring and damping contributions to leg force generation. (*b*) Comparison of step length and stride frequency between elderly and young groups in the experiment and the simulation.

**Figure 8.**
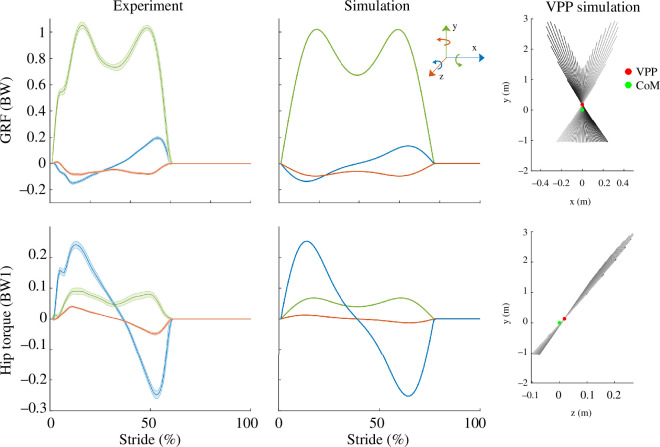
Human and model gait performance comparison. The model’s ability to simulate human behaviour is demonstrated. The first column displays the experimental ground reaction force and hip torque data,while the second column shows the corresponding values generated by the model. Owing to the model’s lackof leg dynamics during the swing phase, the comparison focuses on the hip torque generated by the ground reaction force around the hip joint in both the model and the experiment. The last column presents the sagittal and frontal plane VPP generated by the model.

In the elderly group, the VPP position was observed to be anterior to both the CoM and the VPP position of the young group. This anterior shift of the VPP in the model leads to an increased forward inclination of the trunk. To stabilize the inclined trunk, counterclockwise torques at the hip joint are necessary. However, this causes an increase in the model’s energy with each step, resulting in instability after a few steps. To address this issue and dissipate the excess energy, we introduced a damper in parallel with the leg spring. The effects of the spring and damper are illustrated in figure 7*a*. Consequently, the VPP shift in front of the CoM resulted in a reduction in step length and increase in stride frequency. To compare these simulation results with experiment, we showed step length and stride frequency for experiment in figure 7*b*. The experimental results show significant difference between step length and stride frequency between young and elderly groups (*p* < 0.05). Also, experimental results are in line with the results from simulation (see figure 7*b*).

## 4. Discussion

This study aimed to investigate the presence and characteristics of the VPP in two distinct groups of young adults and elderly individuals walking at varying speeds. VPP reflects the relationship between GRFs, CoP and CoM, offering insights into how each age group manages their overall angular momentum, which is crucial for balance. In the following, we summarize the insights from experimental and simulation studies.

### 4.1. Experimental insights

#### 4.1.1. Influence of age and speed on virtual pivot point quality

The current findings suggest that the quality of VPP in the sagittal plane remains consistently high across both younger and older age groups, irrespective of walking speed. Notably, younger participants displayed significantly higher $R^{2}$ values compared to older counterparts (see table 2). The sagittal-plane GRFs during human walking demonstrate a consistent directional alteration as the CoP transitions from anterior to posterior of the CoM, resulting in the convergence of GRFs at a distinct point [26]. This coordinated pattern contributes to the observed high-quality sagittal VPP in both age groups, indicating that sagittal-plane dynamics play a crucial role in VPP quality. Despite age-related changes, a high-quality VPP persists within the sagittal plane. By contrast, this systematic behaviour is not observed in the frontal plane. The higher $R^{2}$ value in the younger group, especially at lower speeds, may be attributed to active control in the frontal plane. Our observations reveal that VPP quality is significantly lower among the elderly population in the frontal plane. These findings highlight distinct control schemes for whole-body angular momentum control among young and elderly populations, potentially contributing to reduced balance control among the elderly. Although our results do not directly establish a relationship between VPP quality and balance control, it is commonly assumed that VPP in the sagittal plane provides postural stability during bipedal walking [9,34]. However, non-VPP gaits derived from a two-dimensional neuromuscular model based on muscle reflexes demonstrate stable walking patterns without the typical intersection of GRFs, suggesting that VPP may not be necessary for postural stability in locomotion [35]. While emphasis is often placed on the intersection of GRFs in the sagittal plane, the significance of the frontal plane in balance should not be disregarded. Our results indicate differences in frontal plane dynamics between age groups, underscoring the importance of addressing frontal plane dynamics in understanding balance control. Moreover, these findings correspond with previous investigations that underscore the heightened significance of balance control within the frontal plane, particularly when compared to the sagittal plane [18,36,37].

The findings of this study reveal that an increase in speed reduces the quality of VPP in both age group and plane. However, this reduction in $R^{2}$ is negligible in the sagittal plane compared to the frontal plane. These results align with earlier research indicating that with increased speed, dynamic walking stability decreases [38], highlighting the potential link between VPP quality and balance control. Statistical analysis also shows a significant effect of dominant leg on the quality of the VPP which is lower for the dominant leg compared to non-dominant leg. However, this effect is negligible compared to the effect of age and speed (see table 2). This difference could be the result of asymmetries in foot pressure distributions and foot axis angles reported in literature for the dominant and non-dominant legs [39]. Also, another study reported a greater amount of CoP variability in the dominant leg compared to the non-dominant leg in both the anterior–posterior and mediolateral directions during body weight squats, which could potentially contribute to the lower $R^{2}$ value for dominant leg observed in our study [40].

#### 4.1.2. Influence of virtual pivot point position with respect to centre of mass

The position of the VPP with respect to the CoM plays a pivotal role in shaping the body’s angular and translational momentum. It also influences energy distribution [41]. In the sagittal plane, we observed that as walking speed increased, the VPP height demonstrated a notable reduction for both the young and elderly groups. This observation is also consistent with other studies on VPP at different speeds [9,42,43]. This decrease in VPP height results in a decrease in the lever arm of the external force around the CoM, leading to a narrower range for the body’s angular momentum [44]. This velocity-dependent relationship between speed and VPP height might be attributed to heightened angular momentum cancellation arising from the reduction in stance phase duration [44]. Additionally, the reduction in VPP height at higher speeds introduces heightened acceleration and deceleration forces in the fore-aft direction. Moving on to the VPP’s positioning along the *x*-direction, the elderly group exhibited a more pronounced horizontal shift compared to the younger group, indicative of a greater reliance on hip extensors among the elderly (see table 1). This adaptation in VPP positioning directly addresses the redistribution of joint torques and powers, a phenomenon previously reported as an effect of ageing [45].

In the frontal plane, the vertical position of the VPP is higher for the dominant leg, particularly in elderly individuals. This phenomenon may be influenced by factors such as the position of the CoP relative to the CoM, the pelvis angle during the support phase, and the ratio between vertical and mediolateral GRFs. Studies indicate that the dominant leg experiences lower medially directed peak forces during walking, which could explain the higher vertical VPP position in the frontal plane [46]. Our experimental results could make the hypothesis mentioned in [46] stronger that dominant leg plays a larger role in mediolateral balance control, as higher VPP means more body weight support.

### 4.2. Simulation insights

#### 4.2.1. Force feedback is more sensitive

Our simulation findings highlight that impaired leg force feedback yields the most substantial impact on the quality of the VPP, especially in the frontal plane. This simulation result can be linked to the quality of VPP observed in the experimental data in the sagittal and frontal planes. Drawing on these outcomes, it becomes plausible to posit that impaired load receptors could be a major contributing factor to the lower $R^{2}$ observed within the elderly group. According to the literature, load receptors and gravity-related sensorimotor information are important factors in stabilizing human posture [47,48]. For instance, human balance control is disrupted transiently in individuals who experienced extended exposure to low gravity conditions [49]. Additionally, during gait, the strength of leg extensor activation during the stance phase is load dependent [48]. These findings suggest a possible connection between the quality of the VPP and the complex mechanisms involved in human balance control. However, to validate this hypothesis, experimental studies are necessary. Additionally, our research emphasizes the significance of incorporating load-related reflexes into simulation models for a comprehensive understanding of balance control in simulations.

Our experimental results show that VPP quality decreases by increasing speed, which contrasts with our simulation results. The simulations indicate that VPP quality improves with increasing speed at different levels of sensory noise (see figure 5). This discrepancy suggests that factors beyond noisy feedback might contribute to the lower VPP quality. One possibility is delayed sensory feedback. For instance, the age-related increase in H-reflex (Hoffmann reflex) latency indicates a delay in the spinal reflex loop, which has been proposed in the literature as a possible contributor to postural instability [50]. In our model, we could not study the effects of delayed sensory feedback owing to the limitations of the model (see §4.3).

#### 4.2.2. Relationship between virtual pivot point position and walking speed

According to figure 4, increased gait speed shows a reduction in the VPP height within the sagittal plane. This observation prompted an investigation into whether VPP position could serve as a governing parameter for regulating forward speed. To test this hypothesis, our model was subjected to tests involving an increase in VPP height to measure its impact on forward velocity. The outcomes of our simulations suggest that alterations in VPP height exert minimal influence on gait speed, thus leading to the conclusion that VPP height is not a determinative control parameter for modulating speed. The simulation results suggest that the reduction in VPP height is merely a consequence of increased speed, and other mechanisms may also contribute to modulating gait speed. For instance, leg stiffness has been introduced as a factor for modulating gait speed [51]. These results also indicate that VPP height can be modulated without altering gait speed. This implies that the VPP position and upper body dynamics can be used for additional purposes, such as improving gait stability or efficiency, which could be valuable in the design of robots and assistive devices [52].

#### 4.2.3. Altered trunk posture and shifts in leg viscoelasticity

Our findings reveal that the VPP in older adults is positioned anteriorly compared to the VPP observed in young adults in the sagittal plane. Placing VPP anteriorly in the simulation increases trunk forward lean and results in an unstable gait. This trend is consistent with previous research that has highlighted alterations in trunk posture as a common occurrence with advancing age [53]. The gait is unstable in simulation because having an inclined trunk requires the hip joint to constantly generate power to prevent the trunk from collapsing forward. This observation is in agreement with the literature indicating that age causes a redistribution of joint torques and powers, with the elderly using their hip extensors more than younger adults [45]. This generated energy needs to be dissipated throughout each cycle to maintain a consistent energy level in the model. The added leg damper in our model is related to the energetic consequence of having inclined trunk orientation (figure 6). This choice is supported by prior studies that have indicated an age-related increase in leg damping, especially at higher gait speeds [54]. Therefore, the VPP position can give us some information regarding the distribution of joint torques and could be used as a target to design assistive devices and rehabilitation treatments. Shifting the VPP forward reduces step length and increases stride frequency, aligning with experimental data. These gait changes may contribute to the higher metabolic cost observed in elderly individuals.

### 4.3. Modelling and its limitations

Template models offer a convenient means to comprehend the intricacies of walking and explore diverse hypotheses within the confines of a controlled set of variables. However, the act of simplifying the multifaceted aspects of human locomotion into an abstract model comes at the cost of information loss and is inherently constrained. An evident limitation emerged in the form of introducing a delay to sensory information. Notably, the introduction of delay to model parameters, such as leg force, resulted in an augmentation of forward velocity to an extent where the model’s stability was compromised at certain junctures.

## 5. Conclusions

Following a decade since the introduction of the VPP, a central question remains unanswered: does the VPP play a fundamental role in balance regulation? Our findings indicate that the VPP quality can serve as a dynamic metric for discerning differences in gait between young and elderly individuals, particularly within the frontal plane where balance holds the highest significance. Prior research has indicated that dynamic balance decreases with age and speed. In this research, we also showed that the effect of age and speed can be mirrored on the VPP quality. This implies that the quality of the VPP could potentially mirror the quality of balance control. Beyond its metric role, employing VPP control in simulations can also explain the underlying balance strategies individuals adopt, including the influence of leg force feedback. Moreover, these simulations can offer valuable insights for devising assistive devices and clinical interventions aimed at enhancing balance.

The greater VPP quality observed in the younger group suggests that directing GRF vectors towards a VPP could constitute the primary strategy for maintaining balance during a steady gait. Conversely, in the light of the reduced capacity to regulate whole-body angular momentum in the elderly, alternative strategies, such as leg adjustment, might come into play to preserve balance. Most of the studies target the design of assistive systems to improve the balance focus of swing leg adjustment strategies. However, based on the comparison between the young and elderly groups, our results suggest that control strategies based on regulating whole-body angular momentum might have priority over other strategies for balancing during gait [55].

## Data Availability

The dataset and codes for model are available online [56].

## Appendix A. Three-dimensional equations of motion for Bipedal spring-loaded inverted pendulum model with a rigid trunk

The three-dimensional model introduced in §2 and the VPPC controller are described in this section. Let us define the CoM position by (x,y,z) and the trunk angles in the sagittal, frontal, and horizontal planes, respectively by $\phi_{s}$, $\phi_{f}$, and $\phi_{h}$. The model shown in figure 1*b* can be formulated by the following equations:

(A 1)
$$\begin{array}{l} \text{SS:}\left\{ \begin{array}{l} m\ddot{x}={\text{GRF}}_{x}\\ m\ddot{y}={\text{GRF}}_{y}-mg\\ m\ddot{z}={\text{GRF}}_{z}\\ J_{s}\ddot{\phi_{s}}=\tau_{s}+d_{s}({\text{GRF}}_{x}\sin\phi_{s}-{\text{GRF}}_{y}\cos\phi_{s})\\ J_{f}\ddot{\phi_{f}}=\tau_{f}+d_{f}({\text{GRF}}_{z}\sin\phi_{f}-{\text{GRF}}_{y}\cos\phi_{f})\\ J_{h}\ddot{\phi_{h}}=\tau_{h}-F_{lh}l_{h}\frac{\cos(\psi_{h}-\gamma_{h})}{l_{h}+\sin(\psi_{h}-\gamma_{h})} \end{array}\right.\\ \text{DS:}\left\{ \begin{array}{l} m\ddot{x}=\sum_{i}{\text{GRF}}_{xi}\\ m\ddot{y}=\sum_{i}{\text{GRF}}_{yi}-mg\\ m\ddot{z}=\sum_{i}{\text{GRF}}_{zi}\\ J_{s}\ddot{\phi_{s}}=\sum_{i}\tau_{si}+d_{s}\sum_{i}({\text{GRF}}_{xi}\sin\phi_{s}-{\text{GRF}}_{yi}\cos\phi_{s})\\ J_{f}\ddot{\phi_{f}}=\sum_{i}\tau_{fi}+d_{f}\sum_{i}({\text{GRF}}_{zi}\sin\phi_{f}-{\text{GRF}}_{yi}\cos\phi_{f})\\ J_{h}\ddot{\phi_{h}}=\sum_{i=1,2}\tau_{hi}-\sum_{i}F_{lhi}l_{hi}\frac{\cos(\psi_{h}-\gamma_{h})}{l_{hi}+\sin(\psi_{h}-\gamma_{h})} \end{array}\right. \end{array}.$$

The parameters of this model are described in table 3. In equation A 1, the ground reaction forces are:

(A 2)
$$\begin{array}{l} {\text{GRF}}_{xi}=F_{lsi}\frac{x_{h}-x_{fi}}{l_{si}}+\tau_{si}\frac{y_{h}}{l_{si}^{2}}+\tau_{hi}\frac{z_{h}-z_{fi}}{l_{hi}^{2}}\\ {\text{GRF}}_{yi}=F_{lsi}\frac{y_{h}}{l_{si}}-\tau_{si}\frac{x_{h}-x_{fi}}{l_{si}^{2}}-\tau_{fi}\frac{z_{h}-z_{fi}}{l_{fi}^{2}}\\ {\text{GRF}}_{zi}=F_{lfi}\frac{z_{h}-z_{fi}}{l_{fi}}+\tau_{fi}\frac{y_{h}}{l_{fi}^{2}}+\tau_{hi}\frac{x_{h}-x_{fi}}{l_{hi}^{2}} \end{array},$$

**Table 3.** Three-dimensional BTSLIP model parameters. (BTSLIP stands for Bipedal spring-loaded inverted pendulum model with a rigid trunk, VBLA stands for velocity-based leg adjustment.)

name	symbol	units	literature	chosen	reference
mass	m	kg	60–80	80	[37,57]
leg stiffness	k	kN m${,}^{-1}$	16–26	18	[34,58]
leg length	$l_{0}$	m	16–26	1	[34]
angle of attack (sag.)	α	${(}^{\circ })$	71–78	VBLA	[33,59]
angle of attack (fron.)	β	${(}^{\circ })$	$f\,\left(\dot{z}\right)$	VBLA	[33,57,60]
dist. hip-CoM	$r_{h}$	m	0.1	0.1	[32,34]
trunk inertia (sag.)	J	kg m${,}^{2}$	4.8	4.8	[32]
trunk inertia (front.)	J	kg m${,}^{2}$	4.8	4.8	[32]

in which the hip position is defined as follows:

(A 3)
$$\begin{array}{l} x_{h}=x-d_{s}\text{cos}\phi_{s}\\ y_{h}=y-d_{s}\text{sin}\phi_{s}\\ z_{h}=z-d_{f}\text{cos}\phi_{f} \end{array}.$$

Furthermore, the leg force $f_{l}$ is the produced force by the leg spring and damper. We use a bilinear damper as a more realistic model of the leg damping behaviour in running, as described in [59]:

(A 4)
$$F_{li}=k(l_{0}-l_{i})-d{\dot{l}}_{i}(l_{0}-l_{i}).$$

The selected values for the model simulation parameters are stated in table 3. The parameters are chosen for generating a stable walking gait at $1.1\;m\;s^{-1}$. A comparison between experimental data and the results of our simulation model is shown in figure 8.
